# Spectrum and Prevalence of Pathogenic Variants in Ovarian Cancer Susceptibility Genes in a Group of 333 Patients

**DOI:** 10.3390/cancers10110442

**Published:** 2018-11-14

**Authors:** Magdalena Koczkowska, Natalia Krawczynska, Maciej Stukan, Alina Kuzniacka, Izabela Brozek, Marcin Sniadecki, Jaroslaw Debniak, Dariusz Wydra, Wojciech Biernat, Piotr Kozlowski, Janusz Limon, Bartosz Wasag, Magdalena Ratajska

**Affiliations:** 1 Department of Biology and Medical Genetics, Medical University of Gdansk, Debinki 1 St., 80-210 Gdansk, Poland; magda.koczkowska@gumed.edu.pl (M.K.); natalia.krawczynska@gumed.edu.pl (N.K.); alina.kuzniacka@gumed.edu.pl (A.K.); izabela.brozek@gumed.edu.pl (I.B.); bartosz.wasag@gumed.edu.pl (B.W.); 2 Laboratory of Clinical Genetics, University Clinical Centre, Smoluchowskiego 17 St., 80-952 Gdansk, Poland; 3 Department of Gynecologic Oncology, Gdynia Oncology Center, Powstania Styczniowego 1 St., 81-519 Gdynia, Poland; stukan@wp.pl; 4 Department of Gynaecology, Gynaecological Oncology and Gynaecological Endocrinology, Medical University of Gdansk, Kliniczna 1A St., 80-219 Gdansk, Poland; marcin.sniadecki@gumed.edu.pl (M.S.); jdebniak@wp.pl (J.D.); dariusz.wydra@gumed.edu.pl (D.W.); 5 Department of Pathology, Medical University of Gdansk, Smoluchowskiego 17 St., 80-952 Gdansk, Poland; wojciech.biernat@gumed.edu.pl; 6 Institute of Bioorganic Chemistry, Polish Academy of Sciences, Noskowskiego 12/14 St., 61-704 Poznan, Poland; kozlowp@yahoo.com; 7 The Gdansk section of the Polish Academy of Sciences, Jaskowa Dolina 31 St., 80-286 Gdansk, Poland; jlimon@gumed.edu.pl

**Keywords:** ovarian cancer, low-penetrance gene, *BRCA1*/2, PARP1 inhibitor, next-generation sequencing, *CHEK2*, *NBN*, *BARD1*, mismatch repair genes

## Abstract

Constitutional loss-of-function pathogenic variants in the tumor suppressor genes *BRCA1* and *BRCA2* are widely associated with an elevated risk of ovarian cancer (OC). As only ~15% of OC individuals carry the *BRCA1/2* pathogenic variants, the identification of other potential OC-susceptibility genes is of great clinical importance. Here, we established the prevalence and spectrum of the germline pathogenic variants in the *BRCA1/2* and 23 other cancer-related genes in a large Polish population of 333 unselected OC cases. Approximately 21% of cases (71/333) carried the *BRCA1/2* pathogenic or likely pathogenic variants, with c.5266dup (p.Gln1756Profs*74) and c.3700_3704del (p.Val1234Glnfs*8) being the most prevalent. Additionally, ~6% of women (20/333) were carriers of the pathogenic or likely pathogenic variants in other cancer-related genes, with *NBN* and *CHEK2* reported as the most frequently mutated, accounting for 1.8% (6/333) and 1.2% (4/333) of cases, respectively. We also found ten pathogenic or likely pathogenic variants in other genes: 1/333 in *APC*, 1/333 in *ATM*, 2/333 in *BLM*, 1/333 in *BRIP1*, 1/333 in *MRE11A*, 2/333 in *PALB2*, 1/333 in *RAD50,* and 1/333 in *RAD51C*, accounting for 50% of all detected variants in moderate- and low-penetrant genes. Our findings confirmed the presence of the additional OC-associated genes in the Polish population that may improve the personalized risk assessment of these individuals.

## 1. Introduction

Ovarian carcinoma (OC), one of the most common gynecological malignancies, accounts for an estimated 239,000 new cases and 152,000 deaths worldwide annually, with the highest incidence rate in Central and Eastern Europe (11.4 per 100,000) [1]. Although the 5-year relative survival rate for all stages combined increased from 33.7% in 1975 to almost 50% in 2009 [2], OC is still the leading cause of death from cancer in women. Because of non-specific symptoms and the lack of effective screening tests, most individuals are diagnosed with an advanced disease (stage III or IV) with the 5-year survival rate below 30% [3].

Morphologically, the epithelial neoplasms are divided into five main subtypes (serous, endometrial, mucinous, clear cell and transitional cell tumors) that have different genetic risk factors, treatment responses, and prognosis. High-grade serous ovarian carcinoma represents approximately 70% of OC, followed by endometrial and clear cell cancer (10% each) [4]. The first-line treatment includes optimal cytoreductive surgery and platinum-based chemotherapy; however, chemotherapy sensitivity varies widely among individuals, depending mostly on the tumor’s histologic subtype with its grade, as well as the overall cancer stage [5,6].

Since the identification of tumor suppressor genes, *BRCA1* and *BRCA2* [7,8], of which germline pathogenic variants predispose to a significant increase in breast and ovarian cancer risk, several other cancer-related genes have been associated with their pathogenesis and progression. The application of next-generation sequencing (NGS) significantly facilitates rapid screening for new cancer susceptibility genes, and indeed, a number of pathogenic variants in the so-called moderate- and low-penetrance genes, such as *ATM*, *BRIP1*, *CHEK2*, *PALB2* or *BARD1*, have been reported to be correlated with a moderate lifetime risk for breast and/or ovarian cancer [9]. However, so far for OC, these data are still limited, and only the loss-of-function pathogenic variants of the mismatch repair (MMR) genes, that is, *MLH1*, *MSH2*, *MSH6,* and *PMS2*, are clearly classified as OC-susceptibility genes [10].

In this study, we established the prevalence and spectrum of the constitutional pathogenic variants in the *BRCA1/2* and 23 other cancer-related genes that may play a role in the predisposition to OC, in a large Polish population of 333 unselected OC individuals.

## 2. Results

### 2.1. Pathogenic Variants in BRCA1/2

All individuals included in this study had the comprehensive *BRCA1*/*2* molecular screening as a part of our previous study [11]. In total, pathogenic variants were found in 71 cases (*n* = 71/333; 21.3%), with the vast majority of variants located in *BRCA1* (*n* = 60/71; 84.5%), and only eleven in *BRCA2* (15.5%). Forty individuals (*n* = 40/71; 56.3%) were heterozygous for one of the most recurrent pathogenic variants observed in the Polish population, i.e., c.68_69del (p.Glu23Valfs*17), c.181T>G (p.Cys61Gly), c.3700_3704del (p.Val1234Glnfs*8), c.4035del (p.(Glu1346Lysfs*20) and c.5266dup (p.Gln1756Profs*74), all located in *BRCA1* [12,13,14]. The most common pathogenic variants were c.5266dup (p.Gln1756Profs*74) and c.3700_3704del (p.Val1234Glnfs*8), accounting for 31% (*n* = 22/71) and 21.1% of cases (*n* = 15/71), respectively.

Among *BRCA1/2* positive cases, the most common histological subtype was serous OC (*n* = 59/71; 83.1%), followed by low differentiated (*n* = 5/71; 7%) and endometrial (*n* = 3/71; 4.2%) tumors. Clear cell and mesonephroid tumors each were represented in less than 3% (*n* = 2/71; 2.8%) of positive cases.

**Figure 1:**
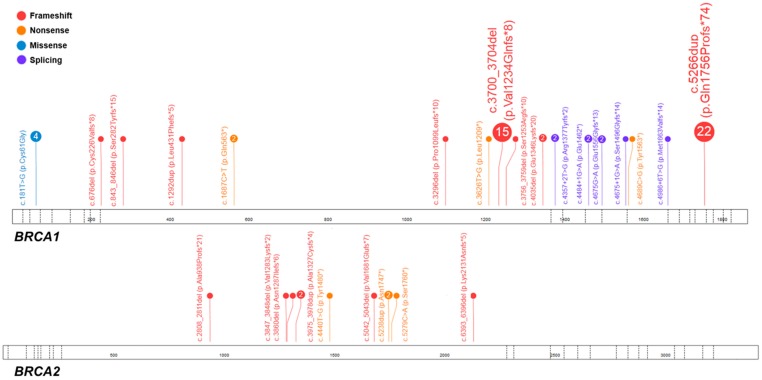
Spectrum of the *BRCA1/2* pathogenic variants identified in the studied cohort of 333 unselected ovarian cancer individuals. Each number in circle corresponds with the total number of individuals heterozygous for a specific variant. The figure was prepared using the ProteinPaint application (^©^Copyright 2015 St. Jude Children’s Research Hospital; 262 Danny Thomas Place, Memphis, TN 38105, USA) [16].

The average age of onset in *BRCA1*/*2* carriers was 58.2 years (range: 27–87), with ten years of difference in diagnosis between *BRCA1* and *BRCA2* individuals (49.3 versus 59 years). All pathogenic variants identified in the *BRCA1* and *BRCA2* genes are displayed in Table 1 and Figure 1.

### 2.2. Pathogenic Variants in Moderate- and Low-Penetrance Genes

To further characterize this cohort, we analyzed 23 additional genes that might play a role in the predisposition to OC. Briefly, we identified 6% of carriers (*n* = 20/333) with 16 different pathogenic variants in one of the tested genes. The most frequently mutated genes were *NBN* and *CHEK2*, with pathogenic or likely pathogenic variants presented in six (*n* = 6/20; 30%) and four individuals (*n* = 4/20; 20%), respectively. Among six cases with the *NBN* pathogenic or likely pathogenic variants, four carried a well-known founder mutation, c.657_661del (p.Lys219Asnfs*16). In addition, we found two different pathogenic variants in the *BLM* and *PALB2* genes, and in *APC*, *ATM*, *BRIP1*, *MRE11A*, *RAD50*, *RAD51C* (each variant observed in a single individual). Additionally, within this cohort, four cases were also carriers of the *BRCA1* pathogenic variants (see details in Table 2).

**Figure 2:**
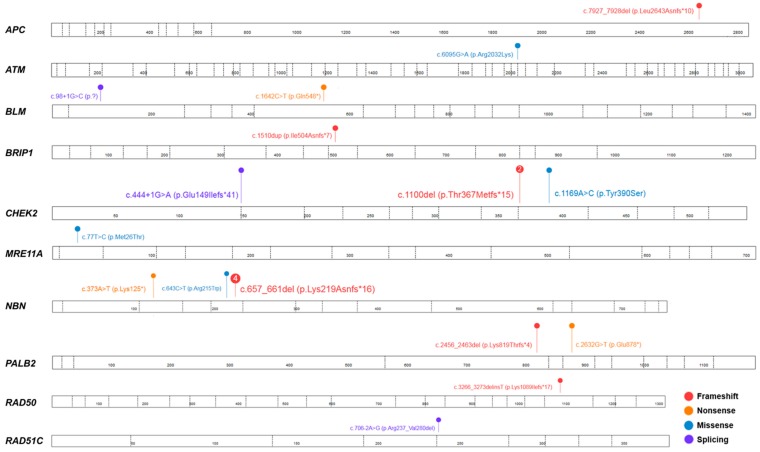
Spectrum of the pathogenic or likely pathogenic variants identified in moderate- and low-penetrance genes in the studied cohort of 333 unselected ovarian cancer individuals. Each number in circle corresponds with the total number of individuals heterozygous for a specific variant. The figure was prepared using the ProteinPaint application [16].

The average age of onset in this group of individuals was 53.9 (range 27–76). All identified variants that were classified as potentially causative (pathogenic or likely pathogenic) are displayed in Table 2 and Figure 2.

### 2.3. Variants of Uncertain Significance (VUS) in Low-Penetrance Genes

Detailed list of all identified VUS is shown in Table S1. The most common alteration was a missense variant in *CHEK2*, c.470T>C (p.Ile157Thr), with a prevalence of 2.7% (*n* = 9/333), including two individuals being recognized as carriers of the *BRCA1* pathogenic variants. The second most frequently observed variant was c.511A>G (p.Ile171Val) in the *NBN* gene, found in six individuals (*n* = 6/333; 1.8%), including two carriers of *BRCA1* and *PALB2* pathogenic variants. Additionally, in two patients, we identified two different variants located in consensus splice site region (c.38-3C>G and c.30G>A) of the *NBN* gene, resulting possibly in an abnormal splicing. However, as no source of RNA was available for these individuals, the missplicing cannot be confirmed.

Finally, we identified an individual classified as a compound heterozygous for *BARD1*, with an in-frame deletion in exon 4, c.1075_1095del (p.Leu359_Pro365del), and a silent mutation c.1977A>G, both variants reside in the *trans* position. The proband, diagnosed with OC at the age of 65, had a positive family history for this type of cancer. The proband’s sister died of OC at the age of 36 over three decades ago; therefore, no biological material was available to perform further genetic testing (Figures S1 and S2).

## 3. Discussion

Constitutional loss-of-function pathogenic variants in the tumor suppressor genes *BRCA1* and *BRCA2* are widely known to confer an elevated risk of breast and/or ovarian cancer, and their mutational screening has become a standard in routine diagnostic practice. It has been estimated that the lifetime risk for developing OC varies from 20% to 50%, depending on the gene in which the pathogenic variant occurs [17]. As only ~15% of OC individuals are heterozygous for the *BRCA1/2* pathogenic variants [18,19,20], the identification of other genes associated with inherited susceptibility to OC is of great clinical importance. So far, several studies aimed at the evaluation of germline alterations in other genes using multi-gene panel testing in a large series of well-characterized OC individuals have been performed, and the potential moderate- and low-penetrance genes have been reported, but their elevated risk of OC is less well characterized [9,10,21,22,23]. Importantly, considering the ethnicity-specific differences in the variants spectrum and frequency, their assessment in all ethnic groups, including the Polish population, is required before making any clinical conclusions. Therefore, in this report, we investigated the prevalence and spectrum of the germline pathogenic and likely pathogenic variants in 25 cancer-related genes through a comprehensive NGS analysis in a large cohort of 333 unselected OC individuals from northern Poland.

**Figure 3:**
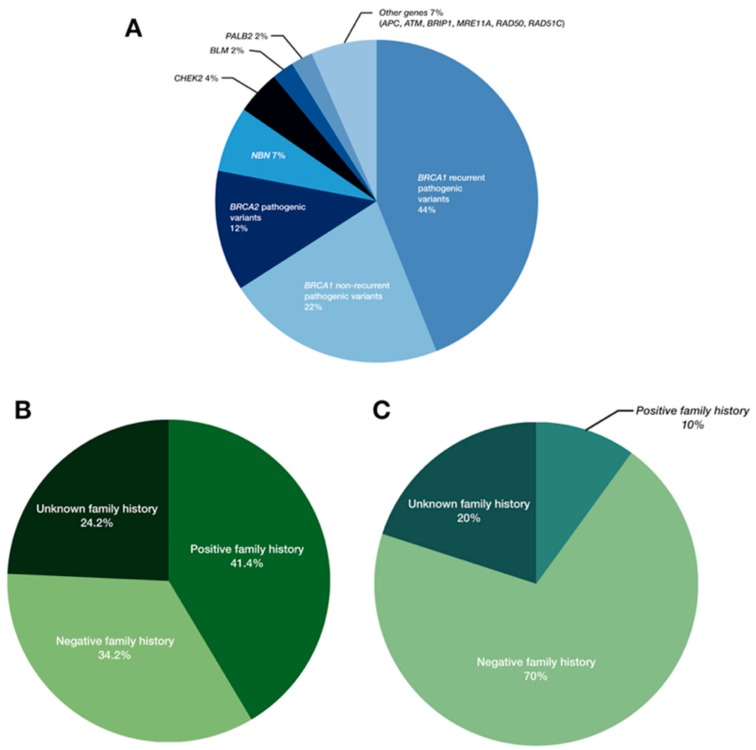
Frequency of pathogenic and likely pathogenic variants in all analyzed genes (**A**), the family history status in the *BRCA1* and *BRCA2* mutation-positive individuals (**B**) and in the individuals heterozygous for the pathogenic or likely pathogenic variants in other breast/ovarian cancer susceptibility genes (**C**).

Overall, we identified 91 pathogenic or likely pathogenic variant in 87 OC individuals (*n* = 87/333; 26.1%;) (Figure 3), in agreement with the previous reports [23,24,25]. Besides the *BRCA1/2* genes, the most frequently mutated were *NBN* and *CHEK2,* with the alterations observed in 1.8% (*n* = 6/333) and 1.2% (*n* = 4/333) of cases, respectively (Table 2). Both genes encode for proteins involved in nonhomologous end joining (NHEJ) or homologous recombination (HR), which are critical mechanisms for the accurate repair of DNA double-strand breaks (DSBs). Particularly, nibrin, the product of the *NBN* gene, regulates the activity of the MRE11A/RAD50/NBN protein complex, while checkpoint kinase 2, the product of the *CHEK2* gene, is activated upon DNA damage, and is responsible for transducing the DNA damage signal to downstream repair proteins, such as p53 [21]. According to the recent National Comprehensive Cancer Network (NCCN) guidelines, germline pathogenic variants in both these genes confer to an elevated risk of breast cancer, with the recommendation of the annual mammogram beginning at the age of 40; for OC individuals, however, these variants are not proven to result in an increased risk of OC, or rather, insufficient evidence have been so far reported [26]. In line with the previous studies, suggesting that the specific *CHEK2* variant c.1100del (p.Thr367Metfs*15) is more prevalent in the Northern and Eastern Europe countries compared with North America [27,28], we identified the presence of this alteration in two cases from the studied cohort (Table 2 and Figure 2). However, both individuals carried simultaneously a pathogenic variant in the *BRCA1* gene; therefore, our findings likely confirmed the previous observations indicating a lack of c.1100del association with an increased risk of OC [29]. Regarding the *NBN* gene, several studies clearly shown the elevated risk of breast cancer with a positive *NBN* testing result, however, this risk assessment was limited to a specific *NBN* pathogenic variant, c.657_661del (p.Lys219Asnfs*16), observed frequently in the Slavic population [30,31]. In this report, variant c.657_661del was present in 4/6 *NBN* mutation-positive individuals (Table 2 and Figure 2). Other potentially causative variants in the *NBN* gene included novel nonsense and missense alterations, c.373A>T (p.Lys125*) and c.643C>T (p.Arg215Trp), respectively; the remaining was previously described in twin brothers with Nijmegen breakage syndrome (MIM: 251260) [32]. All but one individual with a positive *NBN* screening result had a negative family history for cancer, with the negative result of the *BRCA1/2* screening. As there is clear evidence that alterations in this gene predispose to an increased risk of breast cancer, but for determining the risk assessment for OC further studies are required, the clinicians involved in the case of OC individuals should be aware of this potential association.

Several other proteins, coded by one of the following genes: *ATM*, *BLM*, *BRIP1*, *MRE11A*, *PALB2*, *RAD50* or *RAD51C*, interact with BRCA1/2 proteins in the DBs repair process by HR or NHEJ mechanism, and therefore, they are considered as alternative candidates for OC-susceptibility genes. Indeed, it has been reported that the *BRIP1* and *RAD51* pathogenic variants confer at least a 6-fold increased risk for OC [29,33,34], but not for breast cancer, in contrast to the *NBN* and *CHEK2* genes. Although the pathogenic variants in these genes were identified in the current study only in two cases (each in a single individual), from a clinical point of view, these results had a great impact on the management of these individuals, as they should be offered risk-reducing salpingo-oophorectomy (RRSO) at the age of 45–50, in line with the current NCCN recommendations [26]. We also found the pathogenic or likely pathogenic variants in other genes, such as *ATM*, *BLM*, *MRE11A*, *PALB2,* or *RAD50*, occurring sporadically in 1–2 individuals (Table 2), but their role in the predisposition to OC is still unclear [29,35,36,37].

The most striking finding was the paucity of the pathogenic variants in any of the MMR genes, resulting in Lynch syndrome (MIM: 120435), that is, *MLH1*, *MSH2*, *MSH6,* or *PMS2*, that are clearly considered as the major causes of hereditary epithelial OC in addition to *BRCA1/2* alterations, with a lifetime risk of OC estimated at 6–10% [10]. One of the possible explanations might be that OC in Lynch syndrome is mostly of endometrial or clear cell histology [29,38,39], while ~70% of cases in the studied cohort represent the high-grade serous OC subtype. Nevertheless, similar to the *BRIP1* and *RAD51* pathogenic variants carriers, individuals heterozygous for a pathogenic variant within one of the aforementioned MMR genes may consider prophylactic RRSO and/or total abdominal hysterectomy [26]; thus, it is critical to identify early those women to provide them with accurate genetic counseling and personalized surveillance.

In the current study, the pathogenic and likely pathogenic variants in the *BRCA1/2* genes were identified in 18% (*n* = 60/333) and 3.3% (*n* = 11/333) of cases for *BRCA1* and *BRCA2*, respectively, together affecting ~21% of women (Table 1 and Figure 1) who are eligible for the poly (ADP-ribose) polymerase 1 inhibitors (iPARP1) -targeted therapy [40]. Comparing with our preliminary data reported on 134 OC individuals with 20 *BRCA1/2* mutation-positive carriers [11], the prevalence here was higher than previously observed (21% versus 14.9%); however, this is still in line with the recent large-scale study performed on 21,401 families, with the *BRCA1/2* overall frequency of 24% [41]. As expected, the most recurrent pathogenic variants were Polish founder mutations, that is, c.5266dup (p.Gln1756Profs*74) and c.3700_3704del (p.Val1234Glnfs*8), accounting for 6.6% (*n* = 22/333) and 4.5% of all cases (*n* = 15/333), respectively. Indeed, Rebbeck et al. (2018) clearly confirmed based on the analysis performed on 29,700 *BRCA1/2*-mutation positive families that these two alterations are the most commonly-observed variants in Eastern Europe, including Polish population [19].

The natural consequence of comprehensive molecular screening is a growing number of VUS, for which interpretation is often challenging and clinically problematic. In the present study, we described an individual being a compound heterozygous for *BARD1*, with an in-frame deletion in exon 4, c.1075_1095del (p.Leu359_Pro365del), and a silent mutation c.1977A>G, reside in *trans* position (Figures S1 and S2). Both alterations lead to missplicing and overexpression of alternative isoforms, named *gamma* and *eta*, and consequently, to significant telomere instability [42]. Taking into account a positive family history for OC, potential actionability of those variants cannot be excluded.

A considerable percentage of mutation-positive individuals for whom the personal data was available was diagnosed at the age of ≥60 (*n* = 18/85; 21%). Numerous carriers presented a negative family history for cancer, confirming prior observations of the limited significance of age on onset and family history when selecting patients for genetic screening (Table 1 and Table 2 and Figure 3B,C) [11,20,43].

In conclusion, we reported here the spectrum and prevalence of the pathogenic and likely pathogenic variants in OC-susceptibility genes in a Polish population. Through a comprehensive NGS analysis of the high-penetrance *BRCA1/2* genes and 23 additional moderate- and low-penetrance genes in a large cohort of 333 unselected OC women from northern Poland, we estimated the overall ethnicity-specific pathogenic variants’ frequency in these genes at ~26%, including ~21% of the *BRCA1/2* mutation-positive individuals and ~5% of cases with pathogenic alterations in the other cancer-related genes.

Although the mutational screening using multi-gene panel testing have not yet had therapeutic implications, as only the individuals confirmed with an advanced high-grade serous OC and heterozygous for the germline or somatic *BRCA1/2* pathogenic variant are eligible for the targeted therapy with iPARP1 (The Polish National Health Program), the identification of the alterations in other OC-susceptibility genes may significantly improve the personalized risk assessment for OC in these individuals. Patients with *RAD51C* and *RAD51D* pathogenic variants who were enrolled in the ARIEL 3 trial showed a significant response to the treatment, with an average progression-free survival time (PFS) of 16.4 months (range, 5.4–30.4 months) [25]. In addition, reversion mutations leading to regained function of HR-proteins (e.g., BRCA1, RAD51C and RAD51D) in tumor cells result in PARP resistance [44]. These results indicate that the target group of OC patients that may benefit from iPARP therapy is more heterogeneous, and as a consequence, that targeted therapies programs inclusion criteria should be amended accordingly.

The application of this approach will allow early detection in those women with pathogenic variants in the genes that clearly predispose them to an elevated risk of OC, providing them accurate genetic counseling with prophylactic management options. Finally, as several of these OC-susceptibility genes, i.e., *NBN, ATM* or *BRIP1*, are associated with the development of autosomal recessive disorders, such as Nijmegen breakage syndrome, ataxia-teleangiectasia (MIM: 208900) or Fanconi anemia (MIM: 609054), respectively, genetic counselling for carriers of pathogenic variants in any of these genes should also include a discussion of reproductive and prenatal screening possibilities.

## 4. Materials and Methods

### 4.1. Individuals and Sample Collection

The study comprised 333 unselected for age or family history ovarian cancer individuals that were referred to the University Hospital in Gdansk and the Red Cross Hospital in Gdynia between 2007 and 2013.

The histological diagnosis of ovarian cancer was evaluated by an expert pathologist; the most representative subtypes were as follows: serous ovarian cancer, accounting for 70.3% (*n* = 234/333) of cases, followed by endometrial (*n* = 32/333; 9.6%;), mucinous (*n* = 18/333; 5.4%;), undifferentiated (*n* = 18/333; 5.4%;), and clear cell subtype (*n* = 13/333; 3.9%;). The remaining 22 cases included tumors of mixed type (*n* = 8), unspecified tumors (*n* = 5), carcinosarcomas (*n* = 3), *leiomyosarcomas, and adult granulosa* cell tumors (each observed in a single individual). The average age at diagnosis was 58.6 years (27–87).

Informed consent was obtained from all the individuals and the study was approved by the medical review board of the Medical University of Gdansk (NKEBN/399/2011-2012; NKBBN/446/2015).

### 4.2. DNA Extraction

Genomic DNA was extracted from the whole blood using a red-blood-cell lysis buffer followed by standard phenol-chloroform procedure.

### 4.3. Molecular Analysis

All samples enrolled to the study were screened for *BRCA1* (MIM: 113705) and *BRCA2* (MIM: 600185) pathogenic variants using NGS with *BRCA* MASTR assay v1.2 (Multiplicom, Niel, Belgium). Molecular analysis of additional 23 genes, including *APC* (MIM: 611731), *ATM* (MIM: 607585), *BARD1* (MIM: 601593), *BLM* (MIM: 604610), *BRIP1* (MIM: 605882), *CDH1* (MIM: 192090), *CDKN2A* (MIM: 600160), *CHEK2* (MIM: 604373), *MLH1* (MIM: 120436), *MRE11A* (MIM: 600814), *MSH2* (MIM: 609309), *MSH6* (MIM: 600678), *NBN* (MIM: 602667), *PALB2* (MIM: 610355), *PMS2* (MIM: 600259), *PTEN* (MIM: 601728), *RAD50* (MIM: 604040), *RAD51* (MIM: 179617), *RAD51B* (MIM: 602948), *RAD51C* (MIM: 602774), *RAD51D* (MIM: 602954), *STK11* (MIM: 602216), *TP53* (MIM: 191170), was performed using a combination of TruSeq Custom Amplicon assay (Illumina Inc., San Diego, CA, USA) and the HEAT-Seq Oncology Panel (Roche Sequencing), followed by MiSeq targeted re-sequencing at minimum of 99× coverage (Illumina, Inc., San Diego, CA, USA). The cut-off of 20% was applied. The analysis was performed with Illumina Variant Studio Software v3.0 (Illumina Inc., San Diego, CA, USA), Sequence Pilot (JSI Medical Systems, Ettenheim, Germany) and Geneious Software v9.05 (Biomatters Ltd., Auckland, New Zealand). Presence of pathogenic or likely pathogenic variants detected by NGS analysis was confirmed by bi-directional Sanger Sequencing (ABI PRISM 3130, Life Technologies, Inc., Carlsbad, CA, USA). Interpretation of variants pathogenicity was performed based on the American College of Medical Genetics and Genomics (ACMG) recommendations [15].

## 5. Conclusions

In conclusion, we propose obligatory *BRCA1/2* screening in all OC patients in the Polish population. In addition, in the negative cases analysis of the additional cancer-related genes should be considered, including MMR genes, *BRIP1*, *RAD51C*, and *RAD51D*. Unfortunately, given the potential costs of such expanded diagnostics, it will probably need to be limited to the selected group of patients with specific clinical and histopathological characterization.

To establish recommendations for the expansion of mutational analysis (including other cancer-related genes) in OC-individuals, further studies in larger OC series with detailed family histories are required.

## Figures and Tables

**Table 1:** Clinical, histopathological and molecular data of the ovarian cancer individuals heterozygous for the *BRCA1/2* pathogenic variants.

Case No.	Exon/Intron	Variant in Corresponding cDNA	Predicted Amino Acid Sequence	Variant Type	dbSNP ID ^1^	ACMG Classification ^2^	Age, Years	FIGO Stage	Histology	Family History
*BRCA1* (NM_007294.3; LRG_292t1)										
M115	5	c.181T>G	p.(Cys61Gly)	M	rs28897672	Pathogenic (PS3 + PS4 + PM2 + PP1+ PP5)	36	IIIC	serous	-
M296							54	IIIC	serous	+
K199							46	IIIC	serous	-
K221							51	IIIC	serous	-
D166	11	c.676del	p.(Cys226Valfs*8)	F	rs80357941	Pathogenic (PVS1 + PS4 + PM1 + PM2 + PP1 + PP5)	43	ND	serous	-
K187	11	c.843_846del	p.(Ser282Tyrfs*15)	F	rs80357919	Pathogenic (PVS1 + PS4 + PM1 + PM2 + PP1 + PP5)	46	IIIC	serous	-
D18	11	c.1292dup	p.(Leu431Phefs*5)	F	rs80357528	Pathogenic (PVS1 + PS4 + PM1 + PM2 + PP1 + PP5)	42	IIIC	serous	+
D138	11	c.1687C>T	p.(Gln563*)	N	rs80356898	Pathogenic (PVS1+PS4+PM1+PM2 + PP1 + PP5)	49	ND	serous	ND
K100							45	IIB	serous	+
M397	11	c.3296del	p.(Pro1099Leufs*10)	F	rs80357815	Pathogenic (PVS1 + PM2 + PM4 + PP5)	59	IIIC	serous	ND
D53	11	c.3626T>G	p.(Leu1209*)	N	rs786203884	Pathogenic (PVS1 + PS4 + PM2 + PM4 + PP1 + PP5)	59	ND	low differentiated	ND
M22	11	c.3700_3704del	p.(Val1234Glnfs*8)	F	rs80357609	Pathogenic (PVS1 + PM1 + PM2 + PP5)	61	IIIB	serous	ND
M38							43	IIIC	serous	-
M66							52	ND	serous	ND
D3							45	IIIC	serous	+
D23							47	IV	serous	-
D63							55	IIIC	serous	+
D70							48	ND	serous	ND
D71							47	ND	serous	-
D104							56	ND	low differentiated	-
D136							43	IIIB	serous	-
D156							47	ND	serous	-
K65							64	IIIC	serous	+
K125							50	IIIC	serous	-
K152							66	IV	serous	+
K189							63	IIIC	low differentiated	+
K74	11	c.3756_3759del	p.(Ser1253Argfs*10)	F	rs80357868	Pathogenic (PVS1 + PS4 + PM1 + PM2 + PP1 + PP5)	45	IV	serous	+
D15	11	c.4035del	p.(Glu1346Lysfs*20)	F	rs80357711	Pathogenic (PVS1 + PS4 + PM1 + PM2 + PP1 + PP5)	54	IIB	serous	-
K222							ND	IIB	low differentiated	+
M92	13	c.4357+2T>G; r.[4186_4357del];[=]	p.(Arg1377Tyrfs*2)	S	rs80358152	Pathogenic (PVS1 + PS2 + PM2 + PP1 + PP3 + PP5)	48	IIIC	serous	+
M95	13						45	IIIC	serous	-
M395	14	c.4484+1G>A; r.[4358_4484del];[=]	p.(Glu1462*)	S	rs80358063	Pathogenic (PVS1 + PS3 + PP1 + PP3 + PP4 + PP5)	41	IIIC	serous	ND
K201							52	IV	mesonephroid	+
D140	15	c.4675G>A; r.[4665_4675del];[=]	p.(Glu1556Glyfs*13)	S	rs80356988	Pathogenic (PS3 + PM1 + PM2 + PP1 + PP5)	37	IIIC	serous	ND
K73 K108	15	c.4675+1G>A; r.[4485_4675del];[=]	p.(Ser1496Glyfs*14)	S	rs80358044	Pathogenic (PVS1 + PS3 + PM1 + PM2 + PP1 + PP3 + PP5)	44 41	IIIC IIIC	serous clear cell	ND -
D152	16	c.4689C>G	p.(Tyr1563*)	N	rs80357433	Pathogenic (PVS1 + PS4 + PM1 + PP1 + PP3 + PP4)	38	ND	serous	-
D72	16	c.4986+6T>G, r.[4916+1_4916+65ins];[=]	p.(Met1663Valfs*14)	S	rs80358086	Pathogenic (PS3 + PS4 + PM1 + PM2 + PP1 + PP3 + PP5)	47	IIIC	serous	+
M50	20	c.5266dup	p.(Gln1756Profs*74)	F	rs397507247	Pathogenic (PVS1 + PM1 + PP5)	52	IIIC	serous	+
M108							63	ND	serous	ND
M138							65	ND	serous	+
M225							50	IIID	serous	+
M226							51	IIIC	serous	+
M227							58	IIIC	serous	-
M314							46	IIIC	endometrial	+
M323							37	IIIC	serous	+
M368							60	ND	serous	ND
M374							66	ND	serous	ND
M378							36	ND	serous	ND
D9							43	IIIB	endometrial	+
D27							66	IIIC	serous	ND
D66							47	ND	serous	+
D83							50	IIIC	serous	+
D99							ND	IIIC	serous	ND
D105							50	ND	serous	+
D144							56	IIC	serous	ND
D149							40	ND	serous	-
K53							60	IIIC	serous	+
K121							52	IIIB	clear cell	-
K197							43	IIIC	endometrial	-
***BRCA2*(NM_000059.3; LRG_293t1)**										
M164	11	c.2808_2811del	p.(Ala938Profs*21)	F	rs80359351	Pathogenic (PVS1 + PS4 + PM2 + PP5)	81	ND	serous	+
D121	11	c.3847_3848del	p.(Val1283Lysfs*2)	F	rs746229647	Pathogenic (PVS1 + PM1 + PM2 + PP1 + PP5)	ND	ND	serous	ND
D114	11	c.3860del	p.(Asn1287Ilefs*6)	F	rs80359406	Pathogenic (PVS1 + PS4 + PM1 + PM2 + PP1 + PP5)	54	IIIC	serous	+
M8	11	c.3975_3978dup	p.(Ala1327Cysfs*4)	F	rs764689249	Pathogenic (PVS1 + PS4 + PM2 + PP5)	45	IV	serous	+
K183							56	IV	low differentiated	+
K212	11	c.4440T>G	p.(Tyr1480*)	N	rs397507719	Pathogenic (PVS1 + PM1 + PM2 + PP1 + PP3 + PP5)	54	IIIC	serous	+
M178	11	c.5042_5043del	p.(Val1681Glufs*7)	F	rs80359478	Pathogenic (PVS1 + PS4 + PM1 + PM2 + PP1 + PP5)	62	IIIC	serous	-
D160	11	c.5238dup	p.(Asn1747*)	F	rs80359499	Pathogenic (PVS1 + PM1 + PM2 + PP1 + PP5)	51	IIIC	serous	-
K172							55	IIIC	serous	-
K254	11	c.5279C>A	p.(Ser1760*)	N	novel	Pathogenic (PVS1 + PM1 + PM2 + PP1 + PP3 + PP5)	ND	IV	mesonephroid	-
K93	11	c.6393_6396del	p.(Lys2131Asnfs*5)	F	rs397507849	Pathogenic (PVS1 + PM1 + PM2 + PP1 + PP5)	76	IIIC	serous	ND

^1^ RS number based on the dbSNP Database (https://www.ncbi.nlm.nih.gov/projects/SNP/) (as of September 2018); ^2^ Interpretation of variants pathogenicity based on the American the College of Medical Genetics and Genomics (ACMG) recommendations [15], i.e., **PVS1**: null variant (nonsense, frameshift, canonical +/−1 or 2 splice sites, initiation codon, single or multi-exon deletion) in a gene where loss of function (LOF) is a known mechanism of a disease, **PS1**: same amino acid change as a previously established pathogenic variant regardless of nucleotide change, **PS2**: proven de novo (both maternity and paternity confirmed), **PS3**: well-established functional studies, **PS4**: the prevalence of the variant in affected individuals is significantly increased compared to the prevalence in controls, **PM1**: located in a mutational hot spot and/or in critical functional domain, **PM2**: absent from controls, **PM3**: for recessive disorders, detected in *trans* with a pathogenic variant, **PM4**: protein length changes due to in-frame or stop-loss variants, **PM5**: novel missense change at amino acid residue where a different pathogenic missense change has been seen before, **PM6**: assumed de novo, but without confirmation of paternity and maternity, **PP1**: co-segregation with disease in multiple affected family members, **PP2**: missense variant in a gene that has a low rate of benign missense variation, **PP3**: multiple lines of computational evidence support a deleterious effect on the gene or gene product, **PP4**: individual’s phenotype or family history is highly specific for a disease, **PP5**: reputable source reports variant as pathogenic. To classify a variant as pathogenic the following criteria need to be fulfilled: ≥ 2 strong (PS1–PS4) OR 1 strong (PS1–PS4) and ≥3 moderate (PM1–PM6) OR 1 strong (PS1–PS4) and 2 moderate (PM1–PM6) and ≥2 supporting (PP1–PP5) OR 1 strong (PS1–PS4) and 1 moderate (PM1–PM6) and ≥4 supporting (PP1-PP5). **Abbreviations**: **FIGO**: International Federation of Gynecology and Obstetrics; **ND**: no data; **F**: frameshift; **N**: nonsense; **S**: splicing; **M**: missense.

**Table 2:** Clinical, histopathological and molecular data of the ovarian cancer individuals heterozygous for the pathogenic or likely pathogenic variants in moderate- and low-penetrance genes.

Case No.	Exon/Intron	Variant in Corresponding cDNA	Predicted Amino Acid Sequence	Variant Type	dbSNP ID ^1^	ACMG Classification ^2^	Age, Years	FIGO Stage	Histology	Family History	*BRCA1/2* Status
*APC* (NM_000038.4; LRG_130t1)											
M33	16	c.7927_7928del	p.(Leu2643Asnfs*10)	F	novel	Pathogenic (PVS1 + PM1 + PM2)	41	IIIC	serous	-	-
***ATM***(NM_000051.3, LRG_135t1)											
D73	41	c.6095G>A	p.(Arg2032Lys)	M	rs139770721	Pathogenic (PS3 + PM2 + PP3 + PP5)	59	IC	serous/mucinous	-	-
***BLM*(NM_000057.2, LRG_20t1)**											
M66	2	c.98 + 1G>C	p.(?)	S	rs750293380	Pathogenic (PVS1 + PM2 + PP3)	52	ND	serous	-	*BRCA1*: c.3700_3704del
M71	7	c.1642C>T	p.(Gln548*)	N	rs200389141	Pathogenic (PVS1 + PP3 + PP5)	61	IIIC	serous	-	-
***BRIP1* (NM_032043.2; LRG_300t1)**											
K190	11	c.1510dup	p.(Ile504Asnfs*7)	F	rs775735278	Pathogenic (PVS1 + PM2 + PP5)	54	IIB	endometrial	-	-
***CHEK2*(NM_007194.3)**											
M140	3	c.444 + 1G>A, r.[ = ,444 + 1_444 + 2insATAG];[=]	p.(Glu149Ilefs*41)	S	rs121908698	Pathogenic (PVS1 + PS3 + PP3 + PP5)	54	ND	myxoid leiomyosarcoma	ND	-
M374	11	c.1100del	p.(Thr367Metfs*15)	F	rs555607708	Pathogenic (PVS1 + PS3 + PP3 + PP5)	66	IIIC	serous	ND	*BRCA1*: c.5266dup
D104							56	ND	low differentiated	-	*BRCA1*: c.3700_3704del
M113	11	c.1169A>C	p.(Tyr390Ser)	M	rs200928781	Likely pathogenic (PS3 + PM1 + PP3 + PP5)	27	IIIB	serous	-	-
***MRE11A*(NM_005591.3; LRG_85)**											
M42	3	c.77T>C	p.(Met26Thr)	M	rs372068015	Likely pathogenic (PM2 + PM3 + PP1 + PP5)	61	IIB	serous	-	-
***NBN* (NM_002485.4; LRG_158t1)**											
K74	4	c.373A>T	p.(Lys125*)	N	novel	Pathogenic (PVS1 + PM2 + PP3)	45	IV	serous	+	*BRCA1:* c.3756_3759del
K124	6	c.643C>T	p.(Arg215Trp)	M	rs34767364	Likely pathogenic (PS2 + PM1 + PM3)	47	IIIC	serous	-	-
M86	6	c.657_661del	p.(Lys219Asnfs*16)	F	rs587776650	Pathogenic (PVS1 + PS3 + PM2 + PP5)	64	IIIC	serous	-	-
D131							76	ND	serous	ND	-
K135							55	IIIC	serous	-	-
K208							ND	IIIB	serous	-	-
***PALB2* (NM_024675.3; LRG_308t1)**											
M407	5	c.2456_2463del	p.(Lys819Thrfs*4)	F	novel	Pathogenic (PVS1 + PM2 + PP5)	53	IIB	serous	ND	-
K219	7	c.2632G>T	p.(Glu878*)	N	novel	Pathogenic (PVS1 + PM1 + PM2 + PP3 + PP5)	54	IIIC	ND	-	-
***RAD50* (NM_005732.3)**											
M161	21	c.3266_3273delinsT	p.(Lys1089Ilefs*17)	F	novel	Pathogenic (PVS1 + PM2 + PP5)	46	IA	serous	-	-
***RAD51C*(NM_058216.1; LRG_314t1)**											
K237	4	c.706-2A>G, r.[706_837del];[=]	p.(Arg237_Val280del)	S	rs587780259	Pathogenic (PVS1 + PM2 + PP3 + PP5)	ND	IIC	serous	+	-

^1^ RS number based on the dbSNP Database (https://www.ncbi.nlm.nih.gov/projects/SNP/) (as of September 2018); ^2^ Interpretation of variants pathogenicity based on the American the College of Medical Genetics and Genomics (ACMG) recommendations [15], i.e., **PVS1**: null variant (nonsense, frameshift, canonical +/−1 or 2 splice sites, initiation codon, single or multi-exon deletion) in a gene where loss of function (LOF) is a known mechanism of a disease, **PS1**: same amino acid change as a previously established pathogenic variant regardless of nucleotide change, **PS2**: proven de novo (both maternity and paternity confirmed), **PS3**: well-established functional studies, **PS4**: the prevalence of the variant in affected individuals is significantly increased compared to the prevalence in controls, **PM1**: located in a mutational hot spot and/or in critical functional domain, **PM2**: absent from controls, **PM3**: for recessive disorders, detected in *trans* with a pathogenic variant, **PM4**: protein length changes due to in-frame or stop-loss variants, **PM5**: novel missense change at amino acid residue where a different pathogenic missense change has been seen before, **PM6**: assumed de novo, but without confirmation of paternity and maternity, **PP1**: co-segregation with disease in multiple affected family members, **PP2**: missense variant in a gene that has a low rate of benign missense variation, **PP3**: multiple lines of computational evidence support a deleterious effect on the gene or gene product, **PP4**: individual’s phenotype or family history is highly specific for a disease, **PP5**: reputable source reports variant as pathogenic. To classify a variant as pathogenic the following criteria need to be fulfilled: ≥2 strong (PS1–PS4) OR 1 strong (PS1–PS4) and ≥3 moderate (PM1–PM6) OR 1 strong (PS1–PS4) and 2 moderate (PM1–PM6) and ≥2 supporting (PP1–PP5) OR 1 strong (PS1–PS4) and 1 moderate (PM1–PM6) and ≥4 supporting (PP1–PP5). **Abbreviations**: **FIGO**: International Federation of Gynecology and Obstetrics; **ND**: no data; **F**: frameshift; **N**: nonsense; **S**: splicing; **M**: missense.

## Supplementary Materials

The following are available online at http://www.mdpi.com/2072-6694/10/11/442/s1. Figure S1: A pedigree of the family with proband being a compound heterozygote for two *BARD1* variants: an in-frame deletion in exon 4, c.1075_1095del (p.Leu359_Pro365del) and a silent variant in exon 10, c.1977A>G, resulting in the missplicing, [r.159\_1903del], [=] (p.Cys53_Trp635delinsfs*12); Figure S2: Extended analysis result for variants: c.1075_1095del and c.1977A>G; Table S1: Variants of uncertain significance (VUS) identified in the studied cohort of 333 unselected ovarian cancer individuals.
