# Supplementary material for: Spectrum and Prevalence of Pathogenic Variants in Ovarian Cancer Susceptibility Genes in a Group of 333 Patients
Source: Cancers (Basel). 2018 Nov 14;10(11):442. doi: 10.3390/cancers10110442 (PMC6266089; doi:10.3390/cancers10110442)
Supplement: Supplementary file 1 [file cancers-10-00442-s001.pdf]

Supplementary materials: Spectrum and Prevalence of Pathogenic Variants in Ovarian Cancer Susceptibility Genes in a Group of 333 Patients

Magdalena Koczkowska, Natalia Krawczynska, Maciej Stukan, Alina Kuzniacka, Izabela Brozek, Piotr Kozlowski, Marcin Sniadecki, Jaroslaw Debniak, Dariusz Wydra, Wojciech Biernat, Janusz Limon, Bartosz Wasag and Magdalena Ratajska

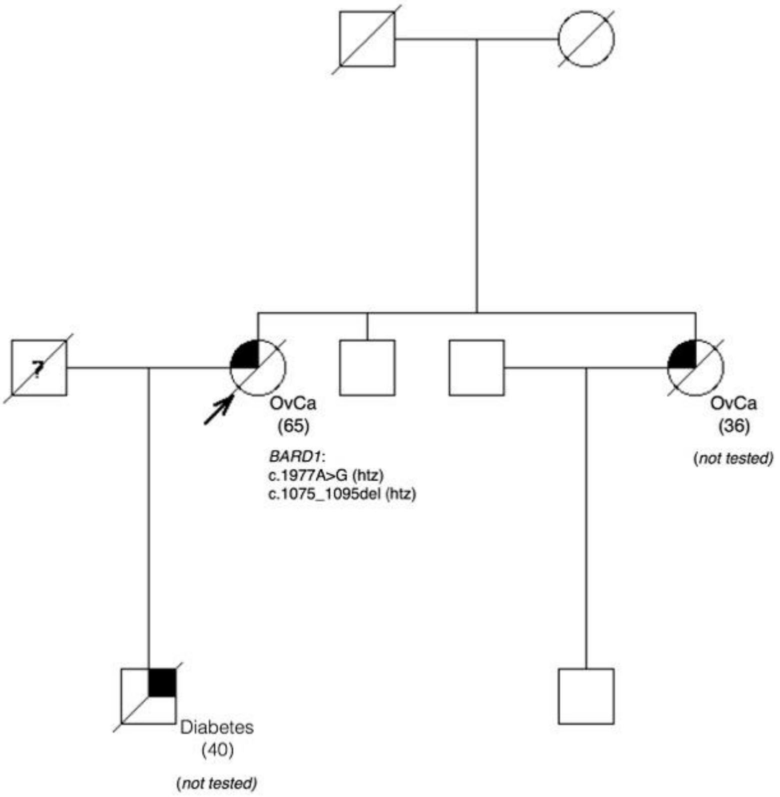

Figure S1. Pedigree of the family (M373) with proband being a compound heterozygote for two *BARD1* variants: an in-frame deletion in exon 4, c.1075_1095del (p.Leu359_Pro365del) and a silent variant in exon 10, c.1977A>G, resulting in the missplicing, r.159_1903del (p.Cys53_Trp635delinsfs*12). Abbreviations: OvCa—ovarian cancer; "→"—proband; (36)—age at diagnosis; "/"—dead.

In order to determine if identified variants affect *BARD1* in *cis* or *trans*, we conducted RT-PCR experiment using a primer specific for 21bp deletion in exon 4 (c.1075_1095del) and second, spanning exon 10 and 11 junction. Agarose gel electrophoresis revealed a band of expected fragment size (959bp), presented exclusively in proband's sample (marked with an arrow) but not in control cDNAs. Consequently, the band was cut from the gel and sequenced. The Sanger sequencing analysis confirmed that variant c.1977A>G resides in trans position to c.1075_1095del.

Sequence of primers used for amplification:

BARD1_Δ21bp_cDNA_frw: 5′-CCTCAGAAAATATACCACCTTCA-3′

BARD1_ex10/11_cDNA_rev: 5′-ATCAAACAGCTTTGGCAACAGC-3′

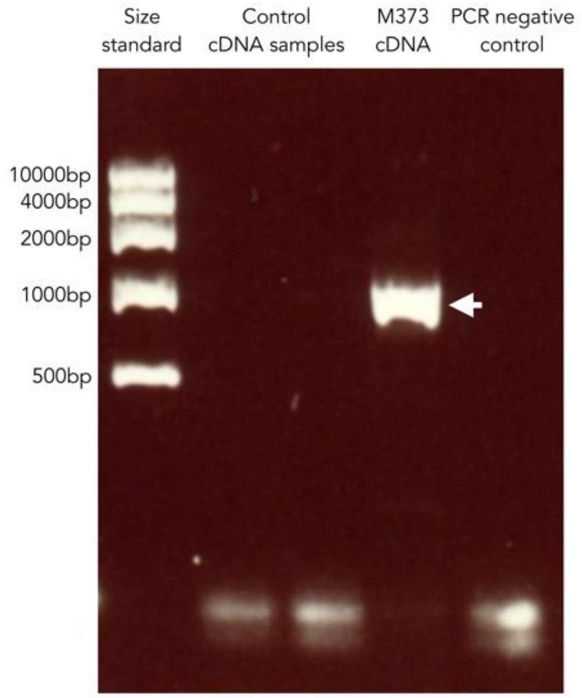

Figure S2. Analysis result for variants: c.1075_1095del and c.1977A>G.

Table S1. Variants of uncertain significance (VUS) identified in the studied cohort of 333 unselected ovarian cancer individuals.

No.	Exon / Intron	Variant in corresponding cDNA	Predicted amino acid sequence	Variant type	dbSNP ID [1]	ACMG criteria [2]
			ATM **(NM_000051.3, LRG_135t1)**			
1	8	c.1066-6T>G	p.(?)	NS	rs201686625	PM2
2	9	c.1229T>C	p.(Val410Ala)	M	rs56128736	PP3+BP6
3	9	c.1792A>C	p.(Ile598Leu)	M	rs56128736	PP3+BP6
4	11	c.1802+4A>G	p.(?)	NS	ND	PM2
5	22	c.3160C>T	p.(Pro1054Ser)	M	rs775095314	PM2+PP3
6	23	c.3402+32_3402+34del	p.(?)	NS	rs545376366	No rules have met the criteria.
7	23	c.3403-34T>A	p.(?)	NS	rs148368017	No rules have met the criteria.
8	28	c.4148C>T	p.(Ser1383Leu)	M	rs141087784	PM2+PP3
9	29	c.4258C>T	p.(Leu1420Phe)	M	rs1800058	PM5+BP6
10	30	c.4600G>A	p.(Val1534Ile)	M	rs745351684	PM2+BP4
11	34	c.5178-7T>C	p.(?)	NS	ND	PM2+BP6
12	37	c.5558A>T	p.(Asp1853Val)	M	rs1801673	PP3+BP6
13	37	c.5674+102A>G	p.(?)	NS	rs4988045	BP4
14	39	c.5918+72A>G	p.(?)	NS	rs3218694	No rules have met the criteria.
15	43	c.6348-54T>C	p.(?)	NS	rs116924981	BP4
16	50	c.7475T>G	p.(Leu2492Arg)	M	rs56399857	PM1+PP3+BP6
17	51	c.7629+107T>A	p.(?)	NS	ND	PM2
18	53	c.7818A>G	p.(Ile2606Met)	M	rs1027959208	PM2+BP4
19	57	c.8396T>A	p.(Phe2799Tyr)	M	ND	PM2+BP4
20	63	c.9104T>C	p.(Leu3035Pro)	M	rs1272213516	PM1+PM2+PP3
			BARD1 **(NM_000465.3)**			
1	1	c.159-261T>G	p.(?)	NS	rs75146556	PP3+BS1

No.	Exon / Intron	Variant in corresponding cDNA	Predicted amino acid sequence	Variant type	dbSNP ID [1]	ACMG criteria [2]
2	4	c.760A>C	p.(Ile254Leu)	M	rs879253984	PM2+PP3+BP4
3	4	c.1075_1095del	p.(Leu359_Pro365del)	D	rs28997575	PM4+PP3+BS1+BP6
4	4	c.1177G>A	p.(Gly393Ser)	M	ND	PM2+PP3+BP4
5	8	c.1811-77A>G	p.(?)	NS	rs76486825	BP4
6	10	c.1977A>G r.[=,159_1903del]	p.(Cys53_Trp635delins fs*12)	S	rs147215925	PS3+PP3+PP5+BP6
7	11	c.2251C>T	p.(Arg751Trp)	M	rs139785364	PM2+PP3
8	11	c.2282G>A	p.(Ser761Asn)	M	rs142155101	PP3+BP4
***BRIP1* (NM_032043.2; LRG_300t1)**						
1	3	c.205+20C>A	p.(?)	NS	ND	PM2+BP4
2	11	c.1622A>G	p.(Asn541Ser)	M	ND	PM2+PP3+BP1
3	13	c.1883G>A	p.(Gly628Asp)	M	ND	PM2+PP3+BP1+BP4
4	19	c.2845C>A	p.(Pro949Thr)	M	ND	PM2+PP3+BP1+BP4
***CDH1* (NM_004360.3; LRG_301t1)**						
1	2	c.163+65G>T	p.(?)	NS	ND	PM2+PP3+BP1
2	2	c.164-8C>T	p.(?)	NS	ND	PM2+PP3+BP1+BP4
3	3	c.194G>T	p.(Arg65Met)	M	ND	PM2+PP3+BP1+BP4
4	5	c.687+66A>C	p.(?)	NS	ND	PM2+PP3+BP1
5	7	c.836C>T	p.(Thr279Ile)	M	rs761269950	PP3+BP1
6	11	c.1711+120G>A	p.(?)	NS	rs535005673	BP1+BP4
7	15	c.2439+6T>C	p.(?)	NS	ND	PM2+PP3+BP1
***CHEK2* (NM_007194.3)**						
1	4	c.470T>C	p.(Ile157Thr)	M	rs17879961	PM1+PP3+PP5+BS1+BP6
2	4	c.507T>A	p.(Phe169Leu)	M	ND	PM2+PP3+BP4
3	11	c.1216C>T	p.(Arg406Cys)	M	rs587782527	PM1+PP3+BP4
4	15	c.1603C>T	p.(Arg535Cys)	M	rs576248104	PM2+ PP3+BP4
5	3′UTR	c.*6G>A	p.(?)	NS	rs775315910	PM2+PP3+BP6 [3]
MRE11A (NM_005591.3)						
1	4	c.315-35C>T	p.(?)	NS	rs201686625	PM2
2	6	c.426C>T	p.(Asp142=)	NS	rs56128736	PP3+BP6
3	8	c.822T>C	p.(Leu274=)	NS	rs56128736	PP3+BP6
4	9	c.862C>T	p.(Arg288Cys)	M	ND	PM2
5	12	c.1327-11G>A	p.(?)	NS	rs775095314	PM2+PP3
6	13	c.1332G>A	p.(Val444=)	NS	rs545376366	No rules have met the criteria.
7	13	c.1475C>A	p.(Ala492Asp)	NS	rs148368017	No rules have met the criteria.
8	13	c.1480G>A	p.(Glu494Lys)	M	rs141087784	PM2+PP3
9	16	c.1868-148G>T	p.(?)	M	rs1800058	PM5+BP6
10	18	c.1995-94G>A	p.(?)	M	rs745351684	PM2+BP4
***MSH2* (NM_000251.2; LRG_218t1)**						
1	6	c.956A>G	p.(Asp319Gly)	M	rs786204185	PM1+PM2+PP3
2	8	c.1378A>G	p.(Met460Val)	M	rs575905950	PM1+PM2+PP3+BP4
***NBN* (NM_002485.4; LRG_158t1)**						
1	1	c.38-3C>T	p.(?)	NS	ND	PM2+PP3+BP4
2	2	c.38G>A	p.(Gly13Glu)	M	ND	PM2+PP3
3	4	c.361G>A	p.(Asp121Asn)	M	ND	PP3+BP4+PM2
4	4	c.481-168C>T	p.(?)	NS	rs104895037	BP4
5	5	c.511A>G	p.(Ile171Val)	M	rs61754966	PP3
6	6	c.703-29C>T	p.(?)	NS	rs104895034	BP4
7	7	c.897-42G>C	p.(?)	NS	rs141172426	BP4
8	14	c.2185-264G>A	p.(?)	NS	rs543500604	BP4
***PALB2* (NM_024675; LRG_308t1)**						

No.	Exon / Intron	Variant in corresponding cDNA	Predicted amino acid sequence	Variant type	dbSNP ID [1]	ACMG criteria [2]
1	1	c.18G>T	p.(Gly6=)	NS	rs587782462	PM2+BP7
2	4	c.756T>A	p.(Thr252=)	NS	ND	PM1+PM2+BP7
3	5	c.2514+21G>T	p.(?)	NS	ND	PM2
RAD50 **(NM_005732.3)**						
1	1	c.122G>A	p.(Gly41Glu)	M	rs1473775948	PP3+BP1
2	8	c.1094G>A	p.(Arg365Gln)	M	rs146370443	BP1
3	9	c.1277A>G	p.(Gln426Arg)	M	rs145428112	BP1
4	9	c.1336A>G	p.(Lys446Glu)	M	rs149217423	PP3+BP4+BP1
5	11	c.1766A>G	p.(Gln589Arg)	M	rs1338200174	BP1
RAD51 **(NM_002875.4)**						
1	5	c.419T>C	p.Leu140Pro	M	ND	PP3
RAD51B **(NM_133509.3)**						
1	3	c.199-5T>G	p.(?)	NS	ND	PM2+BM4
2	7	c.756+118T>A	p.(?)	NS	ND	BP4
3	8	c.853+172T>C	p.(?)	NS	rs192016240	BP4
4	9	c.957+16G>A	p.(?)	NS	ND	PM2
RAD51C **(NM_058216.2; LRG_314t1)**						
1	2	c.376G>A	p.(Ala126Thr)	M	rs61758784	PM1+BP6
2	3	c.572-17G>T	p.(?)	NS	rs193023469	BP6
3	5	c.790G>A	p.(Gly264Ser)	M	rs147241704	BP6
RAD51D **(NM_002878.3; LRG_516t1)**						
1	9	c.812T>C	p.Val271Ala	M	ND	PP3+PM2
STK11 **(NM_000455.4; LRG_319t1)**						
1	5′UTR	c.-117dup	p.(?)	NS	ND	PM2
TP53 **(NM_000546.5; LRG_321t1)**						
1	9	c.949C>A	p.(Gln317Lys)	M	rs764735889	PM1+BP4
2	10	c.1101-49C>T	p.(?)	NS	rs17881850	BP4

[1] RS number based on the dbSNP Database (https://www.ncbi.nlm.nih.gov/projects/SNP/) (as of September 2018); [2] Interpretation of variants pathogenicity based on the American the College of Medical Genetics and Genomics (ACMG) recommendations [1]; [3] Variant c.*6G>A in the *CHEK2* gene potentially affect miRs binding sequence. Abbreviations – M: missense; S: splicing; D: in-frame deletion; NS: not specified.

References

1. Richards, S.; Aziz, N.; Bale, S.; et al. Standards and guidelines for the interpretation of sequence variants: a joint consensus recommendation of the American College of Medical Genetics and Genomics and the Association for Molecular Pathology. *Genet. Med.* **2015**, *17*, 405–424.
